# Differentiated Thyroid Cancer: A Health Economic Review

**DOI:** 10.3390/cancers13092253

**Published:** 2021-05-07

**Authors:** Klaas Van Den Heede, Neil S. Tolley, Aimee N. Di Marco, Fausto F. Palazzo

**Affiliations:** 1 Department of Endocrine & Thyroid Surgery, Hammersmith Hospital, London W12 0HS, UK; n.tolley@imperial.ac.uk (N.S.T.); a.di-marco@imperial.ac.uk (A.N.D.M.); f.palazzo@imperial.ac.uk (F.F.P.); 2 Department of General and Endocrine Surgery, OLV Hospital, 9300 Aalst, Belgium; 3 Department of Surgery and Cancer, Imperial College, London SW7 2AZ, UK

**Keywords:** cost-benefit analysis, economics, medical, thyroid neoplasms

## Abstract

**Simple Summary:**

This review reflects on health economic considerations associated with the increasing diagnosis and treatment of differentiated thyroid cancer. Analysis of different relevant health economic topics, such as overdiagnosis, overtreatment, surgical costs, and costs of follow-up are being addressed. Several unanswered research questions such as optimising molecular markers for diagnosis, active surveillance of primary tumours, and improved risk stratification and survivorship care all influence future healthcare expenditures.

**Abstract:**

The incidence of differentiated thyroid cancer (DTC) is rising, mainly because of an increased detection of asymptomatic thyroid nodularity revealed by the liberal use of thyroid ultrasound. This review aims to reflect on the health economic considerations associated with the increasing diagnosis and treatment of DTC. Overdiagnosis and the resulting overtreatment have led to more surgical procedures, increasing health care and patients’ costs, and a large pool of community-dwelling thyroid cancer follow-up patients. Additionally, the cost of thyroid surgery seems to increase year on year even when inflation is taken into account. The increased healthcare costs and spending have placed significant pressure to identify potential factors associated with these increased costs. Some truly ground-breaking work in health economics has been undertaken, but more cost-effectiveness studies and micro-cost analyses are required to evaluate expenses and guide future solutions.

## 1. Introduction

Thyroid nodules are common and increase with age, especially in iodine-deficient areas [1]. One-third of screened adult cohorts in Europe have a thyroid abnormality on ultrasound [2]. Detected thyroid nodules are investigated to establish a benign or malignant diagnosis and the treatment plan for each of these outcomes is informed by national and international guidelines [3,4]. However, the guidelines rarely include economic considerations.

Differentiated thyroid cancer (DTC), an umbrella term for papillary thyroid cancer (PTC), follicular thyroid cancer (FTC), and Hürthle cell thyroid cancer (HTC) represents approximately 95% of all thyroid cancers [5]. The incidence of DTC has been increasing for the last three decades, but the disease-specific mortality remains stable. Most patients have an excellent prognosis, so the increasing diagnoses have created a vast pool of thyroid cancer survivors that require follow-up. This paper aims to reflect on the health economic considerations associated with the increasing diagnosis and treatment of DTC.

## 2. Epidemiology of DTC

The incidence of DTC is rising worldwide [6]. In 2020, 448,915 new cases of thyroid cancer were estimated, with an age-standardised rate of 10.1/100,000 and 3.1/100,000 in women and men, respectively (Global Cancer Observatory, IARC). In the United Kingdom (UK), where thyroid cancer incidence is lower than most other European countries, it has increased by 68% over the last decade with 3700 new thyroid cancers diagnosed a year. The expectation is a further rise to 11 cases per 100,000 by 2035 (Cancer Research UK). In 2019, 52,070 new cases were estimated in the United States (US) [7]. If the rising trend in incidence is maintained, thyroid cancer should become the fourth most common cancer in the United States by 2030 [8]. Despite the progressive increase in incidence, the disease-specific mortality in the US has increased marginally from 0.40 to 0.46 per 100,000 and can be accounted for by the advanced and dedifferentiated cancers that occur most commonly in an ageing population [9]. In 2020, 43,646 patients died from thyroid cancer (27,740 women and 15,906 men) (Global Cancer Observatory, IARC), almost no change from the 40,000 estimated global deaths in 2012 [6]. These trends of incidence and mortality are seen across the developed world, with pockets of extreme increase in incidence in countries where thyroid screening has been adopted, such as in South Korea [5,10,11]. The rate of incidental DTC however has remained stable in autopsy studies since 1970 [12].

The rising incidence of DTC applies primarily in high-income countries where incidence rates are more than two-fold higher than low and middle-income countries [6]. International comparisons can be difficult due to differences in the reporting and treatment of the disease. However, even within the same country different rates of DTC diagnosis exist, usually coinciding with a different medical ethos, healthcare structure, and/or funding strategy as noted in regions of Belgium and Brazil [13,14,15]. In the US, social-economic group and race are also influential with a higher incidence of thyroid cancer found in white patients with a higher income and health insurance levels [16].

The increasing incidence of thyroid cancer is driven by early-stage DTC without any increase of note in mortality or any increase in the known risk factors [5]. Despite an increase in exposure from medical conditions and their treatments, the overall environmental radiation burden has declined [17,18,19,20].

Iodine deficiency [21] predisposes to goitre and thyroid nodularity which are also risk factors for thyroid cancer diagnosis [5]. A meta-analysis reported a PTC/FTC ratio of 3.4–6.5:1 compared to a ratio of 0.19–1.7:1 in iodine-deficient areas [22]. Chronic iodine deficiency may also be a risk factor for anaplastic thyroid cancer [5,23] but overall, there is no epidemiological overlap between the surge in DTC and iodine deficient areas.

In light of the above considerations, whilst the hypothesis of increased population exposure to known or some unrecognised carcinogens is a potential explanation for the thyroid cancer epidemic, this remains somewhat unlikely.

## 3. Overdiagnosis and Overtreatment

It is probable that the single most important cause of the rising DTC incidence over the last few decades has been the increased detection of asymptomatic thyroid nodularity revealed by the liberal use of thyroid ultrasound. This has unveiled a huge reservoir of mainly benign but sometimes malignant disease. The junction at which diagnosis becomes overdiagnosis is the point at which the identification of disease does not lead to overall population benefit. Overtreatment is an almost inevitable product of overdiagnosis and is observed when a disease is diagnosed and optimally treated but the net result is an unfavourable balance between patient benefit and the overall adverse effects of care. This risk was recognised almost 30 years ago [24] as increasing access to ever-improving imaging techniques replaced medical examination.

The widespread use of neck ultrasound has led to either a preoperative increased detection of early-stage tumours and/or indirectly to thyroid surgery where incidental thyroid cancers, mainly papillary thyroid carcinomas under 10 mm (papillary thyroid microcarcinomas (PTMC)), are found. From 1975 to 2009, the proportion of incidental PTMC has increased from 25% to 39% [25]. In some European countries and the US, 45 to 70% of thyroid malignancies are considered “over diagnosed”, based on studies comparing the expected and observed prevalence of thyroid cancer [26]. This conclusion is also inferred by the rise of early-stage thyroid cancer and the incongruity of there being more cancer with no change in mortality over decades [27]. The alternative explanation, that early diagnosis coupled with excellent treatment has compensated for a real thyroid cancer epidemic, appears less substantiated.

Overdiagnosis and overtreatment are clear generic population-based concepts but are more difficult to define at an individual level since it has not been possible to reliably predict the natural history of an individual PTMC in a specific patient. The early diagnosis of a PTMC will have saved some patients from a late diagnosis and despite the excellent overall prognosis some PTMC may become larger and metastasise or manifest an aggressive clinical behaviour even without enlargement. However, it remains the case that at autopsy after deaths unrelated to thyroid cancer 6.7–16.1% of thyroids present one or more foci of PTMC [12] so that the vast majority of newly diagnosed PTMC is implicitly of no clinical significance. The diagnosis and treatment of PTMC offers no benefit to the patient in the vast majority of cases. Apart from some difficult to identify patients who will benefit, most are exposed to potential morbidity without gain. In addition to the personal risk of surgery, one must consider the psychological effects of a cancer diagnosis that is cancer in name but infrequently in behaviour. A frequently overlooked additional consequence of the surge in DTC diagnoses is the drain on healthcare resources.

The overdetection and treatment of PTMC comes with an economic cost irrespective of whether it is treated surgically or subjected to a surveillance programme. These costs must be balanced against the consequences of a delayed diagnosis in the minority of patients that may come to harm if a timely cancer diagnosis is not made. This argument effectively overlaps with the health economic debate that applies to cancer screening in general. Does thyroid cancer screening fit the cancer screening principle that a presumptive diagnosis of subclinical disease and an early diagnosis improves outcome? [28]. For screening to be effective, the time interval between a disease becoming detectable by the screening tool and the presentation of clinically detectable disease must be shown to be detrimental to the patient [29]. For a screening program to be considered the disease must be common, have an identifiable risk group and the screening tool must be both sensitive and specific. There is no evidence that this applies to thyroid cancer. A Polish group reviewed 4701 patients surgically treated for thyroid cancer [27] with patients divided according to whether the diagnosis was made with a clinical presentation or without symptoms or risk factors. The asymptomatic group predictably presented a lower TNM stage, a lower rate of multifocality, and no characteristics of aggressive clinical behaviour. The use of screening results in the diagnosis of indolent cases and may lead to overdiagnosis and overtreatment. Very few countries have implemented a thyroid ultrasound screening program for thyroid cancer and several governments have now acknowledged the possible detrimental effects of unwarranted neck ultrasound use in asymptomatic patients.

In an attempt to reduce unstructured neck ultrasound screening in the UK, only a thyroid specialist should request a thyroid ultrasound [30]. The American Preventive Service Task Force (USPSTF) recently released its guidelines, in which it strongly recommends against using neck ultrasound for thyroid cancer screening in asymptomatic patients [31]. The impact of changing guidelines was seen in South Korea where screening with neck ultrasound for thyroid cancer became part of a National Cancer Control Program in 1999 [32], creating an epidemic of low-risk PTC. After recognising the morbidity of unnecessary thyroid surgery due to the thyroid screening this practice was discouraged from 2014 with a corresponding decrease in the incidence of thyroid cancer and the number of thyroid operations decreased significantly [33]. However, if patients do not undergo surgery for PTMC, the management dilemmas and costs associated with an active surveillance (AS) programme with repeated clinical review and ultrasounds is also problematic since a reliable predictor of progression is still not available [34,35].

## 4. The Indeterminate Thyroid Nodule

The detection of a thyroid nodule begins a cascade of investigations with neck ultrasound and fine-needle aspiration (FNA) at the heart of the algorithm. Benign cytology should allow patient discharge in most cases. Since it is not always possible to unequivocally exclude malignancy at FNA cytology, surgery may be recommended for these ‘indeterminate’ lesions. However, the cancer rate at final histology after surgery is less than 30% [36]. The problem of indeterminate cytology and 70% of unnecessary thyroid operations may be addressed in some cases with the identification of mutations in molecular panels that are promising [37,38]. Molecular testing, however, adds further expense to the diagnostic workup (£2160–£2880) [39] and usually reduces the risk of rather than guaranteeing the absence of cancer. A recent review of available molecular panels concluded that the more accurate molecular-based test methods are still expensive and restricted to a few, highly specialised and centralised laboratories [40]. Molecular testing is therefore not currently provided by taxpayer-funded healthcare systems since value (benefit/cost) remains unproven.

The cost of mutation panels however needs to be put in the context of the potential saving of unnecessary surgery and the benefit of patient discharge, assuming that this actually occurs. Several cost-effectiveness studies have been performed, comparing lobectomy to genetic testing [37], molecular panel testing [41], or lobectomy and frozen section to total thyroidectomy for thyroid nodules suspicious of cancer [42]. Most studies suggest that a diagnostic lobectomy remains overall preferable to genetic testing as a strategy for ruling out the malignancy of indeterminate thyroid nodules. The conclusions are determined principally by the consequence of “closure” after a hemithyroidectomy versus living under surveillance after using molecular panels which appears to remain the recommendation. A systematic review concluded that the test specificity had to be >68% and the amount of surgery decreased by over 50% for molecular testing to be cost-effective [39]. This health economics model confirmed that molecular evaluation of thyroid nodules with indeterminate cytology could generate positive health outcomes by reducing the rate of unnecessary surgery on benign nodules and may find traction as the costs of the tests decrease.

## 5. Surgery as the Solution?

Thyroid surgery is becoming increasingly expensive. A large population-based study demonstrated increasing patient charges for both inpatient and outpatient elective thyroid surgery, with increasing costs of £644 or 4.31% every year between 2006 and 2014, after controlling for multiple clinical and demographic variables and adjusting for inflation [43]. There is ample evidence that a thyroid lobectomy presents no survival difference compared to a total thyroidectomy in low-risk PTC less than four centimetres in diameter [44]. Hemithyroidectomy has the advantage of retaining natural thyroid function in 80% or more of patients and avoids permanent hypoparathyroidism and its sequelae. The disadvantages of a hemithyroidectomy are the reduction of efficacy of thyroglobulin as a tumour marker, the preclusion of radioiodine as adjuvant treatment, and a higher risk of requiring a second operation for local recurrence. Overall, the 2015 American Thyroid Association guidelines conclude that a lobectomy is an acceptable treatment primarily to avoid the morbidity of total thyroidectomy documented in lower volume practices [3] rather than because it is a better option from an oncological point of view.

Most thyroid surgery in the US and many parts of the world is performed by low-volume general, ENT, and, to a lesser extent, maxillofacial surgeons [45]. Whilst a hemithyroidectomy in low-risk thyroid cancer may offer lower morbidity with unchanged cancer efficacy [46] the cost considerations appear to have taken a back seat. The Quality Adjusted Life Year (QALY) can be used in the assessment of the value of medical interventions [47]. If the Incremental Cost-Effectiveness Ratio (ICER) is applied to the treatment of a solitary thyroid nodule with an FNA biopsy that is ‘suspicious for cancer’ a hemithyroidectomy alone does not appear to be the most cost-effective and appears to be inferior in cost-effectiveness compared to a total thyroidectomy. This calculation is based on a model that includes the accuracy of a frozen section and the rate of injury to the recurrent laryngeal nerve (RLN). Unfortunately, the study failed to factor in the varying rate of malignancy for an FNA biopsy and calculated just 12 months of the life-long hormonal replacement, long-term permanent nerve palsy, and permanent hypocalcaemia. Equally a thorough costing of ultrasound surveillance of the neck was also insufficiently assessed but is likely to add additional cost to the hemithyroidectomy group [42]. As the study was published before the 2015 ATA guidelines, the higher rate of completion thyroidectomy might have altered the cost-effectiveness analysis.

A more recent cost-effectiveness analysis compared total thyroidectomy versus lobectomy for small (2 cm) nodules suspicious for PTC (defined as Bethesda V) [48]. The authors conclude that a total thyroidectomy protocol produced an incremental cost of £1929 and incremental effectiveness of minus 0.24 QALY as compared to the lobectomy protocol. The consecutive sensitivity analysis demonstrated that total thyroidectomy apparently only becomes a cost-effective strategy if the risk of stages III and IV PTC is 82.4% among patients with Bethesda V cytology on preoperative FNA. These counterintuitive findings may be related to the quantification of the risk of morbidity (hypothyroidism, hypoparathyroidism, or unilateral RLN injury) after lobectomy was estimated at up to 50% which is high compared to national registry data [49]. Whether the true cost of follow-up and additional imaging rather than a cheaper nurse led thyroglobulin follow-up have been contemplated was not clearly stated. One feature that is not quantified adequately is the cost of lifelong physician follow-up and frequent office ultrasound in the lobectomy group that is likely to make the surveillance of anything less than a total thyroidectomy more expensive.

## 6. Surgical Technology: A Cost-Effective Addition?

As stated above, the cost of thyroid surgery seems to increase year on year even when inflation is taken into account. In part this may be caused by the increasing use of technology aimed to improve outcomes. The morbidity after thyroid surgery is low when performed by high-volume surgeons [50,51] and the real-world results suggest a gross underreporting of surgical morbidity [52]. Hypoparathyroidism, recurrent laryngeal nerve palsy, and post-operative haemorrhage reduce QoL and add cost to the overall treatment of thyroid cancer [52]. Costs can be reduced with appropriate postoperative hypocalcaemia protocols [53] and the cost of care is consistently lower in high-volume hospitals in the USA mainly due to reduced length of stay but other variations remain unexplained [54]. One possible variable relates to the use of technological adjuncts.

To reduce the morbidity of thyroid surgery, many technical aids have been developed and advocated. These include nerve monitoring devices, vessel sealing devices, autofluorescence technology, and new surgical approaches, such as robotic thyroid approaches and more recently transoral surgery. Naturally, these devices may have advantages to offer in some cases, possibly reducing morbidity or the time of surgery, but always at a cost. The technology adds to the total costs associated with thyroid surgery, as was demonstrated using the Nationwide Inpatient Sample (NIS) database [55] and Premier Healthcare Database [43]. However, often the enthusiasm for new technology has meant that a rigorous cost-effectiveness/value analysis is not performed until the devices have become ingrained in surgical practice. It is clear that some technology may be expensive but more cost-effective than cheaper solutions [56]. For example, energy-based devices for sealing, cutting, and/or secondary haemostasis are now widely used and preferred to the clamp-and-tie approach for this reason [57]. The various technologies (ultrasonic, bipolar, and advanced bipolar) have proven efficacy and safety [57,58,59,60,61] and a pooled cost-effectiveness meta-analysis showed an 8.7% reduction in procedure costs, derived primarily from a reduction in operating time costs, across surgical procedures (*p* = 0.029) [62].

The efficacy data on intraoperative neuromonitoring (IONM) of the RLN and EBSLN in thyroidectomy are now extensive, but it remains controversial whether the use of IONM can reduce the rate of permanent RLN injury in thyroid surgery. Most device users are reluctant to return to thyroid surgery without the device [63] but there has been an attempt to address the value of IONM [64,65,66]. The most recent one evaluated the cost-effectiveness of IONM using a Markov chain model, in the setting of a bilateral thyroidectomy [64]. The ICER between the use and non-use of IONM was £33,401 per QALY with the conclusion that this is an acceptable cost in avoiding bilateral RLN palsy and tracheostomy. However, the cost-utility analysis did not confirm these results completely, reporting visual identification of the RLN led to a cost saving of £129 and £496 per patient, and an improvement of 0.001 and 0.004 QALY, over selective IONM and universal IONM, respectively. It was concluded that if the RLN injury were decreased by 50.4% or more with IONM compared to visual identification, the selective use of IONM in high-risk cases would be the most cost-effective solution [65]. Another analysis failed to demonstrate cost-effectivity in a realistic clinical setting [66]. The use of IONM has however become the standard of care irrespective of the value considerations in most developed countries and has a key role in training to which a price cannot be attached as cannot the value of avoidance of bilateral nerve palsy provided by IONM [67].

Autofluorescence of the parathyroid glands and the use of indocyanine green (ICG) to evaluate their vascularisation is another new surgical technique that recently has been developed [68,69,70]. Time will tell whether this adds value in the event that hypoparathyroidism can be prevented with the associated costs of life-long supplements and end-organ damage including renal impairment.

The costs of novel surgical approaches such as robotic transaxillary thyroidectomy and transoral endoscopic thyroidectomy vestibular approach (TOETVA), which have as the main feature avoiding a neck scar [71], are yet to be evaluated from a health economics point of view. The widespread use of robotic thyroid surgery in Korea has been ascribed to extensive government support, economic interests, and the higher surgical fees associated with the technique [72]. One analysis has compared transoral endoscopic thyroidectomy vestibular approach and transcervical approach thyroidectomy but omitted conventional surgery as a control. Differences in mean variable direct cost for lobectomy and total thyroidectomy were £918 and £745, respectively, due to the longer operating time and different energy-based devices (open versus keyhole) used [73].

## 7. Follow-Up: The Gift That Keeps on Giving?

Current European, British, and American guidelines recommend regular follow-up of DTC after surgery in order to detect early recurrence, supervise TSH suppression, and manage any surgical complications. It is recommended that it be undertaken by a member of the multidisciplinary team according to the established local protocols [3,30,74]. Surgical morbidity after total thyroidectomy adds significantly to the expenses of surgical treatment. Only a few studies have evaluated the cost-effectiveness of different management strategies for vocal fold paralysis and (temporary) hypoparathyroidism [75,76,77].

Lifelong surveillance with hormone replacement or TSH suppression has a cost, and this is increasing cumulatively as the number of thyroid cancers treated with surgery increases coupled with the progressive improvement in generic life expectancy. The low yield of cancer recurrence in all but the most aggressive forms of thyroid cancer has called into question the value of thyroid cancer follow-up, especially three-monthly follow-ups advocated by some in the first year and the Thyrogen^®^-stimulated (Sanofi Belgium, Machelen, Flemish Brabant, Belgium) risk stratification [78].

More than 750,000 thyroid cancer survivors are living in the United States today [25,79,80]. Eighty percent of new thyroid cancer patients are under 65 years of age and the 20-year disease-specific survival is over 90%. The cost of the follow-up of 750,000 patients has to be contextualised with thyroid mortality of just 0.4% of all cancer deaths in the United States [81]. The increasing detection of thyroid cancer and the ageing general population suggest that the thyroid cancer follow-up numbers will continue to rise significantly [82]. The current and projected healthcare-related costs attributable to well-differentiated thyroid cancer care have been studied by Lubitz and colleagues [83]. The total estimated costs associated with WDTC care in 2013 exceeded £1.15 billion in the US alone. The initial treatment including diagnostics, surgery, and adjuvant radioactive iodine (RAI) accounts for £473 million (or 41% of the total annual costs), and an alarming, £428 million (37% of total costs) is taken by the management of the follow-up. There are also hidden costs related to medical practitioner activity and the cost to society as workdays are lost to attend for investigations and doctor visits are not calculated in this budget calculation.

## 8. Active Surveillance of PTMC

Having established that PTMC can be treated conservatively does not mean that the individuals diagnosed with a usually indolent benign behaving thyroid lesion stop being patients. Indeed, avoiding surgery in PTMC may actually be more expensive than surgery as it is replaced by “active surveillance (AS)”.

Japanese data have explored AS for the management of incidentally identified uncomplicated PTMC. Long-term longitudinal follow-up studies in Japan have demonstrated that PTMC can safely be treated conservatively with no significant morbidity and no increase in disease-specific mortality [84,85,86]. Following 1235 patients for up to 227 months with biopsy-confirmed thyroid malignancy showed 0% distant metastatic rates and the small percentage of patients with tumour progression or new lymph node metastases showed excellent outcomes with rescue surgery [35,46]. Given the absence of reliable predictors of which PTMC will remain dormant and which will develop into clinically significant disease, the active surveillance patients are monitored radiologically at variable intervals indefinitely. The cost implication of identifying an indolent thyroid cancer whether followed by surgery or AS is rarely considered, nor indeed the psychological impact of a “cancer” diagnosis that stays with the patient indefinitely.

It has been shown that in an American and Canadian context nonoperative management of PTMC is associated with a modest decrement in QoL. Indeed, a thyroid lobectomy appeared cost-effective and is associated with an ICER of £3192/QALY, well below the study’s willingness-to-pay threshold [87]. Deterministic sensitivity analysis revealed that the cost-effectiveness was highly dependent on the relative disutility of AS, meaning the patient-specific QoL decrement due to AS, as well as on the remaining life expectancy after diagnosis. It remains clear that the diagnosis of PTMC is undesirable both for the patient and the healthcare system except for the minority that develops a true PTC. A recent meta-analysis demonstrated tumour growth in 4.4% of 4156 patients with AS for low-risk PTMC, with only 1.0% developing cervical lymph node metastasis, and 0.04% developing metastatic disease over a pooled mean period of 44 months [88].

## 9. Healthcare Structure and the Growing Cost of Care

Healthcare is funded differently around the world. A broadly speaking socialised healthcare model where treatment is free at the point of access dominates in most of Northern Europe and hybrid schemes with co-payment exist through most of the European Union. Private insurance-based models or self-funded healthcare exists elsewhere. In other words, providing a health episode in some contexts is a societal burden and others an item of service that is associated with a fee and therefore potential profit. The epidemic in thyroid cancer may therefore be seen as a health economic crisis or a wealth opportunity depending on the context in which medicine is practiced.

Studies to explore the economics of the increase in thyroid cancer diagnosis and the associated increase in thyroidectomy rates depending on the health model can be difficult to interpret. In general, cost-effectiveness analyses are hypothetical and present inherent limitations with reproducibility, mainly because of changes in values (probability and cost) over time and the varying model designs [48].

Existing studies on thyroid cancer cost rarely provide a holistic view of the different factors associated with the excess expenditures. Calculations are not contextualised with other cancers and offer an annual estimate of expenditure, without considering the effect of concurrent medical conditions, mental health, and functional status on healthcare expenditures that are paramount to develop future solutions [89,90].

A recent SEER-database study projects the estimated lifetime cost for a hypothetical cohort of individuals with thyroid cancer to be £24,981 per patient, ranging from £24,074 to £42,201 for those with local or metastatic disease respectively. The total cost for an incident cohort of thyroid cancer diagnosed in 2010 was approximately £1 billion and projected to increase to more than £1.7 billion for the 2019 cohort. The total medical cost including diagnosis, treatment, and management for the cohorts diagnosed between 2010 and 2019 is approximately £13.4 billion [91]. Based on the SEER/Medicare data, Boltz et al. estimated the first-year cost for non-metastatic DTC of £12,744 per patient [92]. Berger et al. analysed 183 metastatic thyroid cancers (2003–2005) using a US health insurance claim database estimating the first-year costs to be £43,416 per patient [93]. Another recent study used different US data sources including Medical Expenditure Panel Survey (MEPS) data to estimate the annual direct spending for thyroid cancer to be £3.9 billion in the United States [94]. Lubitz et al, again using the SEER data, conducted a stacked cohort cost analysis from 1985–2013 to estimate current and future healthcare expenditures attributable to well-differentiated thyroid cancer. The current societal costs were estimated to be £1.1 billion in 2013 and predicted to be £2.5 billion in 2030 based on present thyroid cancer incidence trends. The problem is not confined to the US healthcare model.

In Brazil, thyroid cancer increased in incidence from 1.51/100,000 to 4.57/100,000 between 2008 and 2018 with an almost unchanged mortality rate (0.30 to 0.36) [14]. A significant increase in the number of thyroid investigation tools (US, FNA) and treatment/follow-up procedures (surgery, low dose RAI, US) was noted in all geographic regions during the same period. However, procedures related to more aggressive thyroid cancers (neck dissection, high dose RAI) decreased. Costs of thyroid US increased by 91%, FNA costs by 128%, treatment-related costs by 120%. This resulted in immediate costs to the Brazilian public health system of £29.5 million over 8 years. A similar picture has been highlighted in Australia where the estimated economic burden of “excess” thyroidectomies in New South Wales has been demonstrated as significant [95]. The incidence of DTC and total thyroidectomy both doubled between 2003 and 2012, while the mortality rate remained unchanged. The projected increase of 2196 thyroidectomy procedures translated into an additional cost of over £10 million in surgery-related healthcare expenditure alone over a decade. A similar picture has been found in Hong Kong where numbers of thyroidectomies for cancer increased even excluding incidental PTMC [96] with the associated cost implications of £8334 per patient in the first year.

There are of course large differences in healthcare costs in different countries and comparing different healthcare and reimbursement systems is challenging [97]. One study performed a cost-analysis of thyroid cancer care between the United States and France identifying that the US healthcare system spends nearly £7200 more per patient for initial 1-year management of PTC than in France. The main components contributing to this cost disparity were hospital facility (70%) and nuclear medicine (19%) reimbursements, despite a lesser duration of stay and lower use of RAI in the United States. Most studies, unfortunately, fail to consider the costs of lifelong thyroid substitution and monitoring of long-term follow-up. It is indeed probable that the annual follow-up cost matches the original larger outlay of surgery as previously suggested. In a publicly funded healthcare system, this substantial cost impacts the funds available for the care of other pathologies [83].

An American study calculated the excess healthcare expenditures of the community-dwelling thyroid cancer patients compared to non-cancer controls in a propensity score-matched analysis [98]. The yearly average total healthcare expenditures among adults with thyroid cancer were significantly higher compared to propensity score-matched controls (£6896 vs £4194, *p* = <0.001). Similar observations were found in terms of inpatient and outpatient expenditures.

## 10. Do Guidelines Help Control Costs?

Cancer guidelines focus almost exclusively on best care. Cost of care tends to not be considered at all or to be an afterthought years after the implementation. A recent study used a microsimulation model to compare the cost-effectiveness of the revised 2015 ATA guidelines to the 2009 guidelines [99]. One of the aims of these revised guidelines was to reduce the number of total thyroidectomies and surgical complications and, therefore, potentially cost. The study illustrates that the ATA 2015 guideline patient generated greater average QALYs (13.09 vs 12.43) at a lower average cost per patient (£10,612 vs £14,386) [99].

Reducing the cost of care is not only relevant to socialised medicine since it can also have an impact on personal wellbeing and cause insecurities regarding personal wealth with the associated QoL considerations. Financial difficulties are reported by 43% of thyroid cancer survivors and are associated with worse anxiety and depression [81]. A South Korean retrospective cohort study calculated an average personal medical cost of £2547 per patient after diagnosis of thyroid cancer at 2 years [100]. Fighting cancer can be a costly battle and understanding the relationship between patient-reported financial toxicity (FT) and health outcomes can help to support post-treatment cancer survivors. Incorporation of FT assessment into survivorship care planning could enhance clinical assessment of thyroid cancer patients, help address the dynamic and persistent challenges of survivorship, and help identify those most in need of intervention across the cancer care continuum [101].

## 11. Future Considerations

Impalpable thyroid cancers detected by ultrasound have almost always an excellent prognosis. The precursor lesions of DTC are not well-established and recognised pathology lesions, but there is no clear demarcation that differentiates precancerous from cancerous lesions. If these were to be reclassified as an indolent lesion of epithelial origin (IDLE), the need for aggressive therapy and screening would be mitigated [102]. Therefore, the re-definition of these lesions as “papillary lesions in situ” as precursors of malignant tumours might be beneficial in reducing the overdiagnosis and overtreatment of patients with thyroid nodules [103]. Being able to select which patients would develop more aggressive disease will have huge impact on healthcare costs for DTC.

Surgical complications from an often-unnecessary operation, the emotional distress linked to the diagnosis of ‘cancer’, and the stress of follow-up, as well as the financial burden to the individual and society, should not be ignored. The problem affects wealthy countries where the steep rise in thyroid ultrasound and FNA has been driven by access to diagnostic imaging. The reversal or slowing down of this trend requires an understanding of the pathology at all medical levels but is not easy to solve [104]. Education whilst helpful clashes with the realities of defensive medicine where the fear of litigation can intimidate doctors towards more investigations, more interventions, and endless follow-up that transforms every person into a lifelong patient. Future research should be directed towards micro-cost analyses to identify potential factors associated with the increased costs. Cost-effectiveness studies with QALY and ICER calculations should be implemented in future guidelines on treatment, surgical, and follow-up strategy.

Some ground-breaking work in health economics has been undertaken, but more needs to be done on to stem the tide and avert medical bankruptcy. Some changes that have been shown to help are the centralisation of cancer care for an economy of scale and quality assurance that comes from group practice and a multidisciplinary environment.

However, a broader, international approach is required to address the problem of overdiagnosis and overtreatment of thyroid cancer, facilitated by data collection, health economic assessment, subspecialisation, and international health policy that together may find a balance between expenses and clinical benefit for the patient. International societies will have to incorporate health economic considerations into their guidelines. The revised 2015 ATA guidelines stated several research questions that remain unanswered to date: optimising molecular markers for diagnosis, AS of DTC primary tumours, and improved risk stratification and survivorship care. Potential answers could all influence future healthcare expenditures.

## 12. Conclusions

The current thyroid cancer ‘pandemic’ is caused primarily by small PTCs that may have caused no harm in most patients, if left undiagnosed. Regardless of the followed guidelines, healthcare, and insurance system, substantial resources are being used for the diagnosis, treatment, and follow-up of this potentially indolent condition. Increased healthcare costs and spending have placed significant pressure to identify potential factors associated with these increased costs and find solutions. The next decade will determine whether as clinicians we can reverse current trends in the ever-increasing cost of thyroid cancer care.

## 13. Take Home Messages

- The main cause of the rising incidence of DTC incidence over the last decades has been the increased detection of asymptomatic thyroid nodularity revealed by the liberal use of thyroid ultrasound.
- After controlling for multiple clinical and demographic variables, and adjusting for inflation, the cost of thyroid surgery is still increasing.
- The cost of long-term follow-up, active surveillance, and excess healthcare expenditures of the community-dwelling thyroid cancer ‘survivors’ has to be evaluated in light of the different healthcare models.
- Future research should be directed towards micro-cost analyses to identify potential factors associated with the increased costs.
- Cost-effectiveness studies with QALY and ICER calculations should be implemented in future guidelines on treatment, surgical, and follow-up strategy.

## 14. Notes

1. For international comparison between studies and data, all monetary values have been expressed in pound sterling (£) at the time of writing: 1 GBP = 1.39 $US = 1.17 EUR = AUD 1.80.
2. A brief summary contextualising health economic terms and concepts used in this manuscript can be found as Supplementary Material [105,106,107,108,109].

## Supplementary Materials

The following are available online at https://www.mdpi.com/article/10.3390/cancers13092253/s1, Supplementary Material: Summary of Health Economic Terms and Concepts.
