# Supplementary material for: Differentiated Thyroid Cancer: A Health Economic Review
Source: Cancers (Basel). 2021 May 7;13(9):2253. doi: 10.3390/cancers13092253 (PMC8125846; doi:10.3390/cancers13092253)
Supplement: Supplementary file 1 [file cancers-13-02253-s001.zip › cancers-1207451-Supplementary.pdf]

Supplementary Material

Summary of Health Economic Terms and Concepts

Health economics is a branch of economics concerned with issues related to efficiency, effectiveness, values, and behaviour in the production and consumption of health and health care. It draws its theoretical inspiration principally from four traditional areas of economics: finance and insurance, industrial organisation, labour, and public finance [1].

Cost-effectiveness analyses compare alternative treatment in terms of costs and consequences using a structured analytic method. Alternative treatments are always those that improve the patients' health in a long-time perspective; Cost-effectiveness analyses reveal the trade-offs involved in choosing among alternative interventions, with the goal of obtainng the most health possible for the available resources [2].

Micro-cost analysis is a cost estimation method that allows for precise assessment of the economic costs of health interventions. It has been demonstrated to be particularly useful for estimating the costs of new interventions, for interventions with large variability across providers, and for estimating the true costs to the health system and to society [3].

Microsimulation models for health outcomes simulate individual event histories associated with key components of a disease process. These simulated life histories can be aggregated to estimate population-level effects of treatment on disease outcomes and the comparative effectiveness of treatments [4].

The **Quality Adjusted Life Year** (QALY) is a widely used objective measure of the effects of disease burden, including the quantity but also the 'quality of life (QoL)' lived. It is commonly used in health economic evaluations as a means of quantifying the health effect of a medical intervention or a prevention program and ultimately to help payers allocate healthcare resources. If a person lives in perfect health for one year, that person will have 1 QALY (1 Year of Life x 1 Utility Value = 1 QALY) [5].

The QALY can be paired with the unit cost to create the **Incremental Cost-Effectiveness Ratio** (ICER; calculated as GBP£/QALY). ICER is defined by the difference in cost between two possible interventions, divided by the difference in their effect, and represents the average incremental cost associated with an additional unit of the measure of effect [6].

A **Markov Chain Model** is stochastic and models temporal or sequential data. It provides a way to model the dependencies of current information with previous information to create an estimation [7].

References

1. Culyer, A.J.; Newhouse, J.P. *Handbook of health economics*. Elsevier North Holland: Amsterdam, the Netherlands, 2000.
2. Neumann PJ, Sanders GD. Cost-Effectiveness Analysis 2.0. *N. Engl. J. Med.* **2017**,*376*, 203–205. doi: 10.1056/NEJMp1612619.
3. Xu, X.; Grossetta Nardini, H.K.; Ruger, J.P. Micro-costing studies in the health and medical literature: protocol for a systematic review. *Syst. Rev.* **2014**, *3*, 47, doi: 10.1186/2046-4053-3-47.
4. Rutter, C.M.; Zaslavsky, A.M.; Feuer, E.J. Dynamic microsimulation models for health outcomes: a review. *Med. Decis. Making.* **2011**; *31*,10–18. doi: 10.1177/0272989X10369005.
5. Bravo Vergel, Y.; Sculpher, M. Quality-Adjusted Life Years. *Pract. Neurol.* **2008**, *8*, 175–182, doi:10.1136/pn.2007.140186.
6. Leiker, A.J.; Yen, T.W.; Cheung, K.; Evans, D.B.; Wang, T.S. Cost Analysis of Thyroid Lobectomy and Intraoperative Frozen Section versus Total Thyroidectomy in Patients with a Cytologic Diagnosis of "Suspicious for Papillary Thyroid Cancer". *Surgery* **2013**, *154*, 1307–1313; discussion 1313-1304; doi:10.1016/j.surg.2013.06.031.
7. Latouche, G.; Ramaswami, V. *Introduction to matrix analytic methods in stochastic modeling*. SIAM: Philadelphia, PA, USA, 1999.
